# Intestinal Microbiome Changes and Clinical Outcomes of Patients with Ulcerative Colitis after Fecal Microbiota Transplantation

**DOI:** 10.3390/jcm12247702

**Published:** 2023-12-15

**Authors:** Artem Y. Tikunov, Valeria A. Fedorets, Evgenia V. Shrainer, Vitaliy V. Morozov, Valeria I. Bystrova, Nina V. Tikunova

**Affiliations:** 1 Federal State Public Scientific Institute of Chemical Biology and Fundamental Medicine, Siberian Branch of the Russian Academy of Sciences, 630090 Novosibirsk, Russia; arttik1986@gmail.com (A.Y.T.); fedorets@niboch.nsc.ru (V.A.F.); shrayner_ev@cnmt.ru (E.V.S.); doctor.morozov@mail.ru (V.V.M.); v.bystrova@g.nsu.ru (V.I.B.); 2 Department of Obstetrics and Gynecology, V. Zelman Institute for Medicine and Psychology, Novosibirsk National Research State University, 630090 Novosibirsk, Russia

**Keywords:** gut microbiome, ulcerative colitis, fecal microbiota transplantation FMT, clinical outcomes, 16S rRNA gene sequencing

## Abstract

Background and Aims: Ulcerative colitis (UC) is a chronic inflammatory disease that affects many people. One of the possible ways to treat UC is fecal microbiota transplantation (FMT). In this study, changes in the intestinal microbiome and clinical outcomes of 20 patients with UC after FMT were estimated. Methods: FMT enemas were administrated ten times, once a day, and fecal microbiota from three donors was used for each enema. The clinical outcomes were assessed after eight weeks and then via a patient survey. The 16S rRNA profiles of the gut microbiota were compared between three samplings: samples from 20 patients with UC before and after FMT and samples from 18 healthy volunteers. Results: Clinical remission was achieved in 19 (95%) patients at week 8. Adverse events occurred in five patients, including one non-responder. A significant increase in average biodiversity was shown in samples after FMT compared to samples before FMT, as well as a decrease in the proportion of some potentially pathogenic bacteria. Conclusion: The efficacy of FMT for UC treatment was confirmed; however, the duration of remission varied substantially, possibly due to different characteristics of the initial microbiota of patients. Targeted analysis of a patient’s microbiome before FMT could increase the treatment efficacy.

## 1. Introduction

The widespread use of next-generation sequencing (NGS) technologies has provided a detailed characterization of a huge variety of microbial communities, especially bacterial communities associated with the human body. Currently, the human gut microbiota is considered as a dynamic system influenced by the host organism and external factors [1,2]. It is known that the main components of the normal human intestinal microbiota are members of the Firmicutes and Bacteroidetes phyla, although the core group of bacterial species does not coincide in different healthy individuals [3] and various variants of the normal microbiota composition can ensure the stable functioning of these complex microbial communities [4]. An imbalance in the gut microbiota, driven by external or internal factors, can lead to pathological conditions, including not only inflammatory and oncological diseases of the gastrointestinal tract, but also immune system disorders, type 2 diabetes, vascular disease, and even brain dysfunctions [5]. One of the possible ways to influence the intestinal microbiota, along with the use of antibiotics, probiotics, and prebiotics, is fecal microbiota transplantation (FMT)—the introduction of intestinal microbiota from a healthy donor into a patient’s intestinal tract.

It is supposed that the use of FMT had been practiced in China since BC, and in modern times, this procedure was first performed in the treatment of a patient with enterocolitis in 1958 [6]. Recently FMT has been successfully used to normalize the intestinal microbiota in various diseases, including chronic inflammatory bowel diseases [7,8,9,10]. The highest effectiveness of FMT has been shown in pseudomembranous colitis associated with *Clostridioides difficile* [11,12,13,14]. A rapid improvement in patients’ conditions and the high efficiency of FMT (more than 80%) in this disease has been confirmed in multicenter randomized clinical trials [15,16], and now it is recommended to use FMT after the second or third episode of *C. difficile*-associated colitis in the USA and European countries [17]. There is evidence that FMT can also be useful in ulcerative colitis (UC). The effectiveness of FMT for the treatment of UC varied from 20 to 92% in different studies [18,19]. In randomized clinical trials, remission was recorded at the level of 25–27%, which was significantly higher than the placebo effect [20,21,22]. 

The pathogenesis of UC is unclear; it is believed to be complex and mediated by disturbances in the composition of the intestinal microbiota, genetic predisposition of the patient, and environmental factors such as diet, smoking, and physical activity [19]. As for the nutrition factor, the increase in the incidence of UC in recent years is often associated with the spread of the “western” dietary pattern, with an elevated intake of total fat, red meat, and sugar [23]. It is considered that diet can influence the intestinal mucosal barrier both directly and through modulation of the microbiota composition [24]. As in *C. difficile*-associated colitis, the biodiversity of the gut microbiota in UC is significantly decreased, with reduced numbers of Bacteroidetes and Firmicutes, whereas the numbers of Proteobacteria and Actinomycetes increase [25]. There have been cases of the detection of *C. difficile*, *Helicobacter pylori*, *Salmonella* spp., and enteroinvasive *Escherichia coli* in the microbiota of patients with UC [26,27,28,29]. It is still unknown whether UC is the result of an impaired immune response to the normal microbiota or it is a normal immune response to an imbalance in the gut microbiota [19,30]. Despite the apparent obviousness, the mechanisms of the positive effect of FMT in UC are not entirely clear. It is supposed that the effectiveness of FMT is associated with an increase in the diversity of the intestinal microbiota, which leads to an increase in the abundance of “beneficial” bacteria and prevents colonization of the intestine by pathogenic bacteria [19,31]. However, there are other possible mechanisms involved in this process, including the influence of virobiota, the effect of the patient’s immune system, and the introduction of regulatory high- and low-molecular-weight compounds [31,32]. In addition, the list of bacterial species that participate in the microbiota normalization has not yet been determined. 

In this study, changes in the gut microbiomes of 20 patients with UC after FMT were analyzed based on profiling of the 16S ribosomal RNA gene in samples obtained before and after treatment. In addition, the outcomes of the FMT were estimated. 

## 2. Materials and Methods

### 2.1. Study Desing

This was a non-randomized pilot study evaluating the efficacy of an FMT enema in the treatment of patients with UC. The study was based on the analysis of the outcomes and gut microbiota composition of patients before and after FMT using 16S ribosomal RNA profiling. In addition, the microbiota compositions in patients before and after FMT were compared with those in healthy volunteers. Three, six, twelve, and twenty-four months after treatment, patients were interviewed about their health status. All patients and volunteers provided informed consent to the ongoing study and anonymous data processing. The pilot study was conducted in accordance with the Declaration of Helsinki and approved by the Local Ethics Committee of the Autonomous Non-Commercial Organization “Center of New Medical Technologies in Akademgorodok” (protocol #1; 18 January 2018).

### 2.2. Healthy Volunteers and Donors Patients 

Volunteers were young healthy people without chronic diseases (including autoimmune diseases), who had not been infected and/or hospitalized for at least six months and did not use antibiotics during this period. Fecal samples from volunteers were tested for the presence of the infectious agents *Salmonella* spp., *Shigella* spp., enteroinvasive *Escherichia coli*, and *Cryptosporidium* spp. using routine microbiological methods, as well as for helminths and their eggs using microscopy. *C. difficile* toxins A and B were detected using the immunochromatographic rapid test (Toxin A + B (Clostridium difficile) DUO, Vedalab, Alençon, France). In addition, the DNA of *Shigella* spp., enteroinvasive *Escherichia coli*, *Salmonella* spp., and *Campylobacter* were tested using PCR with hybridization fluorescence detection of amplification products (Amplisence OKI Bacto-Screen-FL, Moscow, Russia), and nucleic acids of rotaviruses A, noroviruses I and II, and adenoviruses F were screened for using the same method (Amplisence OKI Viro-Screen-FL, Moscow, Russia). All volunteers were additionally screened for *H. pylori*.

Volunteers who expressed a desire to become donors underwent further examination, which included general and biochemical blood tests, as well as blood ELISA for the presence of lamblia, toxocara, opisthorchia, ascaris, and trichinella. In addition, the absence of the syphilis causative agent, HIV-1 and HIV-2, and hepatitis B and C viruses was confirmed using standard kits. 

### 2.3. Patients

Adult patients diagnosed with UC were involved in the study. The inclusion criteria for patients in the study were as follows: patients of any gender aged from 18 to 70 years; informed consent for FMT; the duration of chronic UC was more than one year; confirmed diagnosis of moderate or moderate to severe UC. The severity of UC was determined on the basis of the Mayo scale, taking into account the frequency of defecation, the presence of blood/mucus in the stool, the state of the intestinal mucosa (determined endoscopically), and the general assessment by a gastroenterologist. When the score on the Mayo scale was 3–5 or 6–9, the UC was considered moderate or moderate to severe, respectively. The diagnosis was confirmed on the basis of a fecal calprotectin level analysis, fibrocolonoscopy (with anesthesia) data, and histological examination of biopsy specimens taken from different parts of the colon and ileum. In accordance with the patient’s roadmap, each patient underwent a general blood test, biochemical analysis (total protein, urea, and bilirubin content), and determination of the concentration of C-reactive protein. All patients were screened for *Clostridioides difficile* or *Helicobacter pylori.* Then, the patient was referred for an electrocardiogram, a consultation with a therapist, and a gastroenterologist. (The anesthesiologist examined the patient before the colonoscopy.) In addition, stool samples from each involved patient were examined for all pathogens listed in Section 2.2 before treatment. The criteria for excluding patients from the study were their age (under 18 and over 79); pregnancy; confirmed diagnosis of irritable bowel syndrome or Crohn’s disease; the presence of *C. difficile*, *H. pylori*, or the above bacterial and viral infections. 

### 2.4. FMT Enema Preparation and Application

The course of FMT included ten enemas, one enema per day. The original donor material had been stored at −84 °C. For each procedure of FMT, the material from three donors was mixed in equal amounts. For each enema, a total of 90 g of stool samples from three donors (30 g from each) was suspended in 250 mL of sterile saline (0.9% NaCl) and the obtained suspension was homogenized using a household mixer at low speed. Then, the mixture was filtrated at least three times to remove large fecal lumps and residues. Each time, the product was freshly prepared 40–60 min before the FMT procedure and stored at room temperature and then, at 35 °C (last 30 min). The volume of each infusion was 190–200 mL. 

### 2.5. Clinical Outcomes

Clinical remission in this study was defined as a frequency of soft/normal stools < 3, the absence/significant decrease in the amount of blood in the stool, a Mayo score < 2, and the absence of or reduction in abdominal pain (score ≤ 1) at week 8 after FMT [33]. A clinical response was recorded when the reduction/disappearance of the following symptoms was observed: abdominal pain, frequency of loose stools, and blood in the stool. In addition, the Mayo score should have decreased by two or more.

### 2.6. Sequencing

Stool samples from patients were collected 1–2 days before FMT and 12–14 days after FMT. A 50 mg aliquot of each fecal sample was suspended in 300 µL of 0.9% NaCl and centrifuged for 10 min at 2000 rpm. Total DNA was purified from 100 μL of clarified cell suspension using a kit for DNA extraction from tissue and blood cells (BioLabMix, Novosibirsk, Russia), with the addition of lysozyme to increase the efficiency of DNA extraction from Gram-positive bacteria. Then, amplification of the 16S rRNA gene fragment (containing variable regions V3–V4) was carried out using the extracted DNA as a template, with fusion primers (NEB-FF 5′-ACACTCTTTC CCTACACGACGCTCTTCCGATCTCTACGGGAGGCA GCAG-3′, NEB-FR 5′-GTGACTGGAGTTCAGACGTGT GCTCTTCCGATCTGGACTACCGGGGTATCT-3′) and high-precision polymerase Q5 (New England Biolabs, Ipswich, MA, USA). The amplification products were purified on AMPure XP magnetic beads (Beckman Coulter, Brea, CA, USA). Enrichment of the obtained amplicons, adding barcodes and adapters for further sequencing on the MiSeq, was performed using Q5 polymerase and a dual index set of oligonucleotides (New England Biolabs, Ipswich, MA, USA), according to the manufacturer’s instructions. The resulting libraries were again purified on AMPure XP magnetic beads (Beckman Coulter, Brea, CA, USA); the DNA concentration was measured using a Qubit dsDNA HS kit (Life Technologies, Carlsbad, CA, USA). According to the measurement results, the libraries were pooled so that their concentrations were approximately equal. Sequencing was performed on a high-performance MiSeq sequencer with an MiSeq reagent kit v2 2 × 250 cycles (Illumina, San Diego, CA, USA).

### 2.7. Data Analysis and Statistics

The sequencing results were analyzed using the QIIME 2 software package v. 2022.8 [34]. At the first step, the reads were filtered by quality using the q2-dada2 plugin [35]. In addition to filtering by quality, DADA2 combines reads obtained from forward and reverse primers and also removes chimeric sequences. The resulting unique sequences (features) were aligned using MAFFT [36] (via q2-alignment) for further phylogenetic analysis with fasttree2 [37] (via q2-phylogeny). The rooted phylogenetic tree obtained by this method was used for alpha diversity analysis. To determine the minimum sampling depth required for analysis, a graph reflecting the number of features depending on the sampling depth was constructed. The Shannon index was calculated using the q2-diversity plugin. The significance of differences between the Shannon indices on the group level was assessed using the Kruskal–Wallis H test. The beta diversity was also computed using the q2-diversity plugin; unweighted UniFrac was used as a distance metric [38]. Pairwise comparison of groups’ distances was performed via permutational multivariate analysis of variance (PERMANOVA) [39]. The features were mapped to the Silva database 138 99% OTUs full-length sequences [40] to determine their taxonomic position via the classify-sklearn method [41], which is included in the q2-feature-classifier [42]. Significant differences in the taxonomic composition between the samples were identified using the ANCOM method included in the q2-composition plugin [43]. The obtained graphs were processed using the Dokdo library v. 1.16.0 [44], Python 3.10.10 (pandas 1.5.3, statannote 0.2.3, matplotlib 3.7.1, seaborn 0.12.2), and R 4.3.2.

## 3. Results

### 3.1. Patients and Volunteers

**Figure 1:**
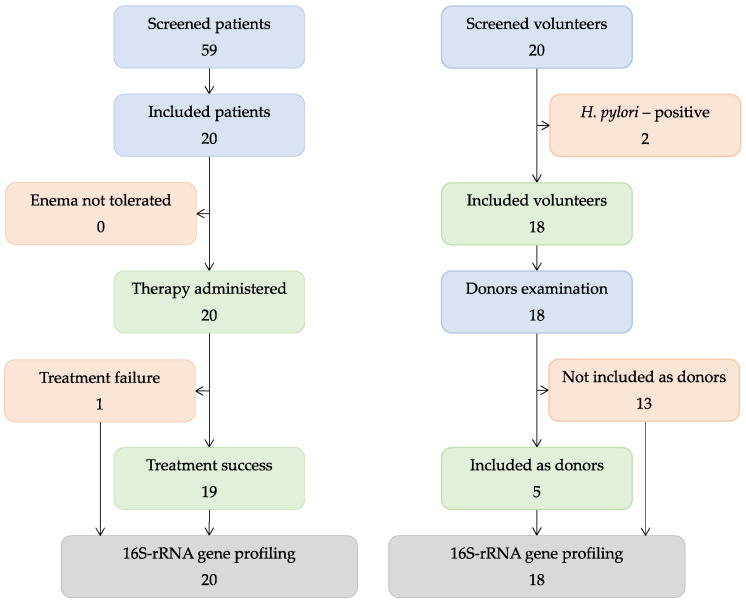
Study scheme.

A total of 59 patients were recruited in the study. After examination, 20 patients (27–57 years old) were enrolled (Figure 1). Before FMT, all patients received 5-aminosalicilate, several used steroids, and no one had antibiotics. The patients’ baseline characteristics are shown in Table 1. All of the enrolled patients received FMT in 2018–2019. Of the 20 volunteers, 2 were not included in the study because of positivity for *H. pylori*. Based on the tests performed, five donors were selected from the volunteers. Their fecal samples were within the sampling from volunteers. 

### 3.2. Clinical Outcomes

All the patients who participated in the study tolerated enemas; so, 20 patients were included in further examinations. The first examination of patients after FMT was performed after two weeks. From the 20 enrolled patients, 19 patients (95%) achieved a clinical response (Table 2). Among the nineteen responders, four patients (21%) had side effects during the FMT treatment: fever (n = 2), nausea (n = 1), and bloating (n = 2). Only in one patient (#13) out of the twenty improvement was not observed. Notably, this patient had several adverse events, namely, fever, diarrhea the day after the third FMT, and abdominal discomfort. At week eight, clinical remission was observed in 12 of the 19 responders (63%). Other responders (27%) had a clinical response but did not achieve clinical remission as the frequency of stool was more than 3 times a day and sometimes they had blood in the stool and felt abdominal pain, although weaker than before FMT. After treatment and the follow-up period (two weeks), all patients were prescribed prescriptions with an individual list of supportive medications, and recommendations on diet and an acceptable lifestyle.

Since most patients were nonresident and came from far away for FMT, it was not possible to assess their endoscopic disease activity further; so, evaluation of the effectiveness of FMT was carried out by interviewing patients by phone or the internet. There was no response from six patients, including the non-responder, and one patient demanded money for answering questions. Among thirteen successfully interviewed responders, nine patients reported clinical remission 12 weeks after FMT; 1 more patient showed improvement. Later, the number of patients with remission decreased; however, clinical remission was observed in four patients even after two years (Table 2). Two out of thirteen interviewed patients who had a clinical response after FMT, but did not achieve clinical remission, reported an improvement that lasted for four months (patient #15) and more than four years (patient #4). Notably, patient #4, who did not achieve clinical remission after FMT (the frequency of stools per day decreased slightly, although the blood in the stool disappeared and abdominal pain became weaker but did not completely disappear), later reported that the condition improved. More than four years after FMT, this patient reported that the frequency of stools was ≤3, there was no blood in the stool, and abdominal pain had completely disappeared. It can be concluded that a long-term clinical remission occurred in this case. Two more patients (#6 and #14) reported that despite the positive clinical response, their condition then worsened. Thus, the effectiveness of FMT among the interviewed patients substantially varied. Notably, four out of eleven patients who had a subsequent relapse of UC associated it with external factors, namely, severe stress, diet violation, surgery and a triple course of antibiotics because of pneumonia, and preparation for sigmoidoscopy. It is important to mention that all patients involved in the study were asked to follow a special diet. Only one patient who had a relapse of the disease connected it to diet violation, but this happened after a 36-month remission (Table S1). Other patients denied violation of the diet.

Importantly, the duration of remission did not depend on the severity of the initial condition of patients with UC. Thus, the average initial Mayo index was 3.4 for patients with remission > 18 months and ~3 for the patient who did not respond to treatment and patients with a clinical response that lasted ≤8 weeks.

### 3.3. Sequencing Results

Three groups of fecal samples were investigated: 18 samples from healthy volunteers (HV), 20 samples from patients with UC before FMT (UC-bef), and 20 samples from the same patients after FMT (UC-aft). DNA isolated from fecal samples was used to construct 58 libraries of 16S rRNA gene fragments. These fragments included the V3 and V4 variable regions commonly used for taxonomic classification of bacteria [45,46]. The results of the library sequencing are shown in Table 3.

**Figure 2:**
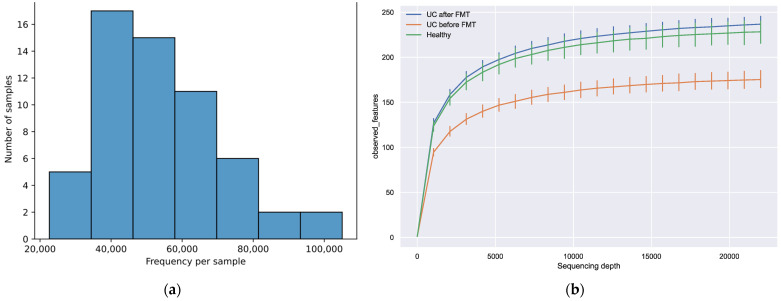
(**a**) Distribution of filtered sequences among the samples. (**b**) The dependence of the observed features number on the sampling depth. The maximum depth was chosen according to the minimum number of reads per sample obtained after filtration.

The number of sequences that passed the filter and were not chimeric ranged from 22,621 to 105,051 (median value—53,557) (Figure 2a). A total of 3679 unique sequences were obtained from all 58 libraries after combining the filtered reads, varying from 1794 to 1959 in different groups. More than 90% of the filtered reads had a length of more than 400 nucleotides, with a total length of the studied gene region of about 460 nucleotides. To determine how fully the obtained data reflect the actual diversity of the microbiota in the studied samples, we built a graph showing the number of identified features depending on the sampling depth (Figure 2b). The maximum depth was chosen according to the minimum number of reads per sample after filtration. The tending of the plots towards a plateau indicates that increasing the sampling depth would not lead to significant changes in diversity; therefore, all obtained data were appropriate for further analysis.

### 3.4. Comparative Analysis of Microbiota Biodiversity in Healthy Volunteers and Patients with UC before and after FMT

**Figure 3:**
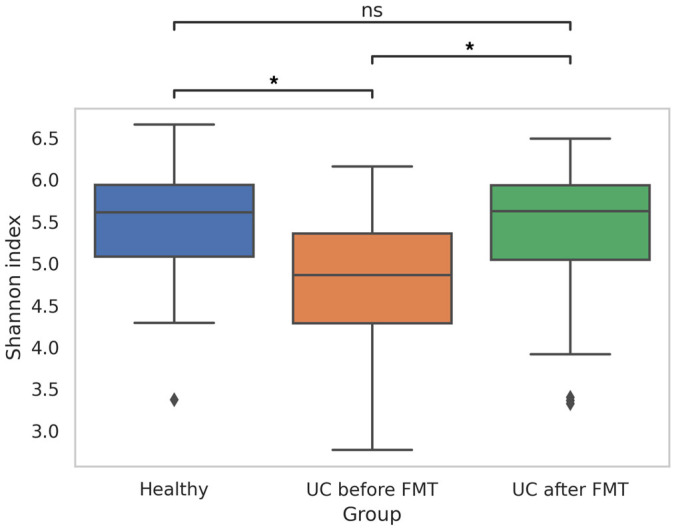
Shannon index, reflecting the biodiversity of sequences in samples obtained from healthy people and patients before and after FMT (* *p*-value ≤ 0.05; ns—no significant differences). Diamonds under the graphs’ whiskers reflect the outliers.

Analysis of the obtained data showed differences in the biodiversity of the bacterial communities in patients before and after FMT and healthy people. Thus, the Shannon index for samples from UC-bef was significantly lower than for samples from UC-aft (*p*-value = 0.025), and also significantly lower than for samples from HV (*p*-value = 0.021) (Figure 3). Pairwise comparison of the Shannon indices for samples from one patient before and after FMT also showed statistically significant differences in all cases; however, in fifteen patients the biodiversity of microbial communities increased and it decreased in five patients (Table S2). Notably, all patients who had a decrease in the Shannon index after FMT initially had a fairly high microbiota biodiversity (the Shannon indices for these patients before the procedure were >5).

These results mainly correlate with the results of other researchers, indicating a reduced biodiversity of the intestinal microbial communities in patients with UC [47,48,49]. Also, they confirm the possibility of increasing the gut microbiota biodiversity after FMT. It should be emphasized that in the present study, we compared the initial microbiome of patients and the microbiome after the first FMT procedure. According to the published data, a statistically significant increase in microbiota biodiversity is not recorded in all patients even after several FMT procedures [50].

**Figure 4:**
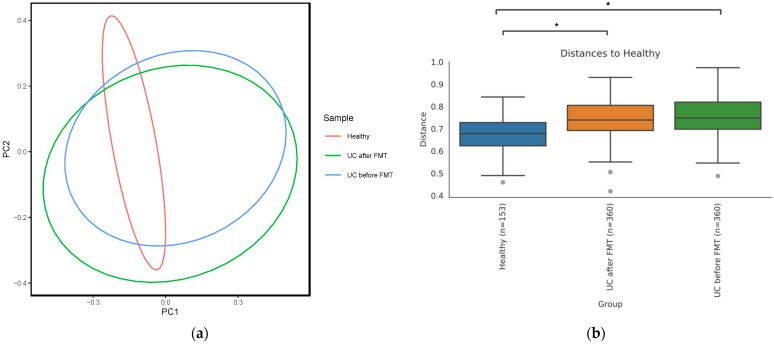
(**a**) PCoA plot based on unweighted UniFrac distance matrix reflecting microbiome beta diversity of healthy people and patients before and after FMT. (**b**) Distances between samples calculated using PERMANOVA test (* *p*-value ≤ 0.05). Grey points under the graphs’ whiskers reflect the outliers.

Analysis of the beta diversity showed that samples from different groups formed noticeably overlapping clusters (Figure 4a). Notably, the beta diversity in samples obtained from patients before and after FMT was more heterogeneous than in the healthy group. 

Permutational multivariate analysis of variance (PERMANOVA) showed that samples within a group of healthy volunteers were significantly more similar to each other than to samples from other groups according to an unweighted UniFrac distance matrix (Figure 4b). At the same time, an analysis using weighted UniFrac did not identify a significant difference in the microbiota diversity between groups, which means that the microbiota of patients with UC both before and after FMT was diverse at the level of poorly represented taxa, while the most prevalent taxa were common for all three groups.

### 3.5. Comparative Analysis of the Microbiota Taxonomic Composition in Healthy Volunteers and Patients with UC before and after FMT

**Figure 5:**
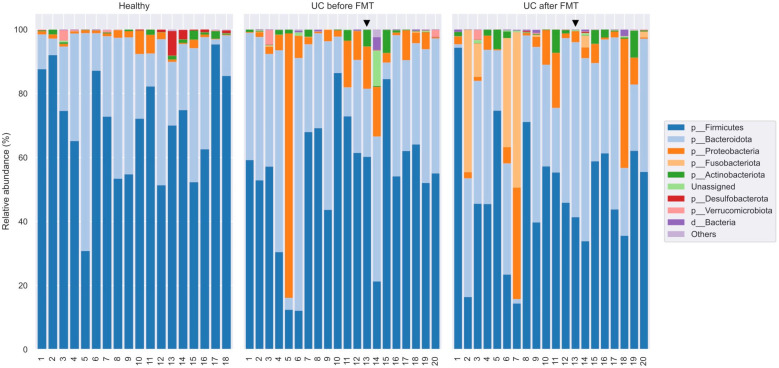
Taxonomic classification of OTUs at the phylum level based on the Silva v.132 (full) database. Black triangle shows the patient who had no clinical response to the therapy.

According to the results of the classification in the libraries from HV, on average, 99.95% of the sequences were taxonomically assigned to the bacteria domain. For libraries from UC-bef and UC-aft, these values were 99.19% and 99.78%, respectively. Only in two libraries, the proportion of unclassified sequences exceeded 1%; both of them belonged to the UC-bef group. In these two samples, the archaeal sequences from the genera *Woesearchaeales* (phylum Nanoarchaeota) and *Methanobrevibacter* (phylum Euryarchaeota) were found; however, the share of such sequences did not exceed 0.36%. In total, sequences of 13 phyla of bacteria were identified, the main ones of which were Firmicutes, Bacteroidota, and Proteobacteria (Figure 5).

The analysis of the taxonomic composition indicated that members of the phyla Firmicutes (73.2%; 30.5–94.6%), Bacteroidota (23.5%; 1.8–68.3%), and Proteobacteria (1.7%; 0.13–6.9%) dominated in samples from healthy volunteers (Figure 5). The next most common phyla were Actinobacteria (0.9%, 0–3.3%) and Verrucomicrobia (0.24%, 0–3.4%). It is known that the microbiota of healthy people consists of permanent and transient species belonging to more than 17 phyla, including Firmicutes (~70%), Bacteroidetes (~30%), Proteobacteria (<5%) Actinobacteria (<2%), Fusobacteria, and Verrucomicrobia (<1%) [51]. Thus, the bacterial community of the HV at the phylum level corresponds to that described for other populations of healthy people.

In samples from UC-bef, on average, 53.8% (from 11.6% to 86.4%) of the detected sequences belonged to the Firmicutes, 32.3% (from 4.4% to 64.4%) belonged to the Bacteroidota, and 9.2% (from 0.4% to 81.3%) belonged to Proteobacteria. Despite the apparent differences in the proportions of these phyla between healthy volunteers (HV) and patients (UC-bef), these differences were not significant. It is noteworthy that there was a tendency of increased occurrence of Proteobacteria in patients, and that in some patients the proportion of Proteobacteria was >10%. A statistical comparative analysis of the diversity using ANCOM showed a significant decrease in the proportion of the Desulfobacterota (on average 0.03% instead of 0.38% in HV) and Synergistota phyla in patients with UC. As for the latter, the sequences of this type were found only in one library from patients with UC and amounted to 0.0006%, while in HV they were found in seven out of eighteen libraries and averaged 0.038%. Notably, there was a significant increase in the proportion of the Fusobacteriota phylum in UC-bef (0.06% on average versus 0.0004% in HV), as well as the proportion of unclassified sequences.

**Figure 6:**
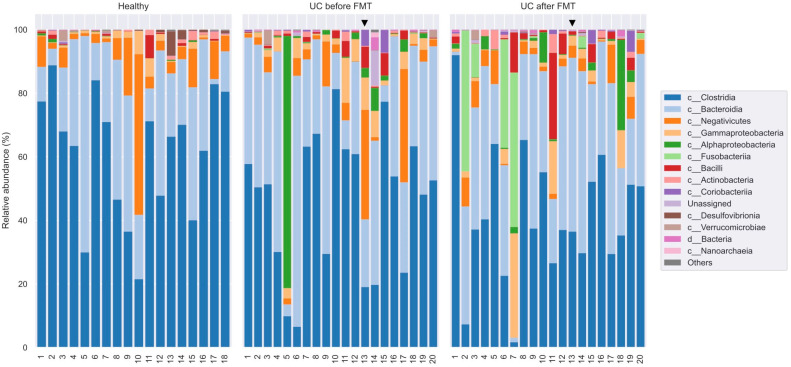
Taxonomic classification of OTUs at the class level based on the Silva v.132 (full) database. Black triangle shows the patient who had no clinical response to the therapy.

As for the samples from UC-aft, on average 49.3% (from 8.9 to 94.4%) of the sequences identified in them belonged to the Firmicutes phylum, 33.7% (from 1.7 to 60.7%) to the Bacteroidota phylum, and 6.2% (from 0.7 to 31.5%) to the phylum Proteobacteria. It should be noted that despite substantial differences in the biodiversity between UC-bef and UC-aft, there were no significant differences in their taxonomic composition. However, a significant increase in the proportion of the Fusobacteriota was found in five patients after FMT: while before the procedure the proportion of these phylum did not exceed 0.3%, after the procedure it increased up to 52.1%. Analysis at other taxonomic levels showed that all sequences of this phylum belonged to the same genus, *Fusobacterium*. The results of taxonomic classification at order, family, and genus level are shown in Figure 6 and Figures S1 and S2, respectively). In addition, four out of five samples that showed a decrease in biodiversity after FMT also had an increased proportion of *Fusobacterium*. This may indicate the possible invasiveness of these bacteria. It should be noted that the pro-inflammatory and oncogenic effect of some *Fusobacterium* on the human intestine has been previously shown [52]. The ratio of the Firmicutes and Bacteroidota phyla is also an indicator characterizing human gut microbiota. In this study, the ratio Firmicutes/Bacteroidota was 3.11 for HV and 1.56 for UC-bef.

The proportions of major bacterial classes representing different phyla are shown in Table 4. The Firmicutes phylum was substantially represented by sequences from the class Clostridia (Figure 4). Representatives of the genera *Faecalibacterium* and *Subdoligranulum* (order Oscillospirales; family *Ruminococcaceae*) and *Roseburia* (order Lachnospirales; family *Lachnospiraceae*) dominated among them. These bacteria are known to be responsible for the digestion of a wide range of carbohydrates, including starch and inulin, with the formation of butyrates [48,53]. Sequences of the classes Negativicutes and Bacilli were also represented. It should be noted that despite the relatively low occurrence of *Bacillus* spp. and *Lactobacillus* spp., their proportion in two patients after FMT increased substantially (by 3.5 and 11 times, respectively). However, there were no significant differences in the representation of the listed taxa between the three groups of samples.

**Figure 7:**
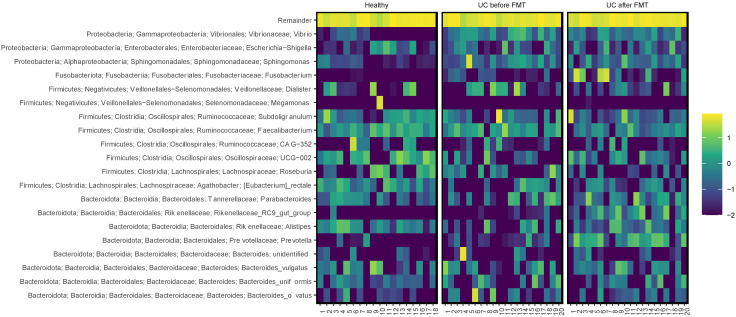
Heatmap showing relative abundance of the most common bacterial genera in samples. The scale reflects the decimal logarithm of the taxon percentage in the sample.

The proportion of sequences belonging to the Bacteroidota phylum in the samples from UC-bef was not reduced and amounted to about a third of all sequences, which differs from the observations of other researchers [48]. This phylum was mostly represented by sequences of the Bacteroidia class, namely, *Bacteroides* spp. and *Prevotella* spp. A comparative analysis revealed a significant increase in the proportion of the genera *Rikenellaceae* and *Prevotella* in patients after FMT, not only compared to patients before the procedure, but also relative to healthy volunteers (Figure 7). It is known that most of the *Bacteroides* in the human intestine are capable of degrading a variety of plant polysaccharides [54], with *Bacteroides* spp. predominating in the intestinal microbiota of Westerners, and *Prevotella* spp. predominating in the microbiota of populations with a plant-based diet [55].

Note that the proportion of Proteobacteria sequences in the samples collected before FMT exceeded that for healthy people. The proportion of *Sphingomonas* spp. belonging to the class Alphaproteobacteria turned out to be significantly increased (Figure 7). Members of this genus accounted for 53% of the total microbiota diversity in one of the patients before FMT; after the procedure, their proportion decreased to 0.03%. It is important to emphasize the potential pathogenicity of the genus *Sphingomonas* for humans [56]. In addition, in patients with UC before FMT, the proportions of the genera *Vibrio* and *Halomonas*, belonging to the class Gammaproteobacteria, were significantly increased. Many members of the *Vibrio* genus are human pathogens and cause severe diseases [57]. The role of the genus *Halomonas* in the human intestine is not clear; their halotolerance has been established [58]. Also, sequences of *Proteus* spp. were found in one sample, and in five samples *Stenotrophomonas* spp. were found; however, their share did not exceed 0.1%. It should be noted that after FMT, the content of the listed Proteobacteria sequences in the samples significantly decreased, to 0.02% or less.

In addition to sequences of pathogenic Proteobacteria, sequences of pathogenic Firmicutes were found in some samples from the group UC-bef. Thus, sequences of *Staphylococcus* spp. (0.1%) were found in one sample, *Streptococcus* spp. in sixteen samples, *Peptostreptococcus* spp. in three samples, and *Enterococcus* spp. (~0.1%) in six samples. As in the case of pathogenic Proteobacteria, the proportion of these sequences after FMT in the corresponding samples substantially decreased.

### 3.6. Clinical Outcomes and Comparative Microbiome Compositions

Next, we compared the microbiome compositions of patients who achieved a clinical improvement at least 12 weeks after FMT and patients whose condition worsened after FMT (#13) or 12 weeks after FMT (#6 and #14). No correlation between a decrease in the alpha diversity and the absence of a positive effect of FMT was found. Notably, there was a significant increase in the proportion of *Fusobacterium* in the microbiota after FMT in patients who reported no improvement (Figure 7). In patient #6 this increase was especially noticeable. Thus, while *Fusobacterium* sequences were not detected before the procedure, they accounted for 34.1% after FMT. In patient 14, the proportion of *Fusobacterium* increased from 0% to 3.6%. However, patients #2 and #3, who reported improvements after FMT, also had a significant increase in this genus: from 0.16% to 44.3% and from 0.37% to 10.5%, respectively. At the same time, no increase in the prevalence of the *Fusobacterium* genus was observed in the non-responder (patient #13). Unfortunately, we could not obtain an answer about the condition of patient #7 after the procedure, in whom the increase in the proportion of this genus was most noticeable (from 0.1% to 48.6%).

Also of interest is the decreased prevalence of the *Lachnospiraceae* family in patients #6 and #14, whereas in most other patients, the number of sequences belonging to this family increased after FMT. The members of the family produce short-chain fatty acids (SCFAs), which are necessary for the functioning of intestinal epithelial cells, and also stimulate the differentiation of regulatory T cells responsible for preventing autoimmune reactions. It has been previously shown that a decrease in SCFA production due to a decrease in the prevalence of *Lachnospiraceae* family members can lead to recurrent UC in patients [59]. In addition, patients #6 and #14 had a significantly increased number of archaeal sequences before the procedure. This decreased after FMT, but remained significantly elevated compared to other patients. Also, these patients showed an increase in the content of the genera *Sutterella*, *Alistipes*, *Oscillibacter*, *Colidextribacter*, *Succinivibrio*, *Negativibacillus*, and *Mogibacterium* after FMT. On the contrary, an increase in *Succinivibrio*, *Veillonella* and archaeal sequences was observed in patient #13 with treatment failure. Previously, other researchers have shown that bacteria of the genera *Alistipes* and *Colidextribacter* can have an anti-inflammatory effect on the human intestine, as well as an antitumor effect in patients with colorectal cancer [60,61]. It is believed that bacteria of the *Succinivibrio* genus also have a beneficial effect on the condition of the human intestine and are able to break down fiber [62]. On the contrary, *Oscillibacter* have been shown to increase intestinal permeability and have a pro-inflammatory effect [63]. An increase in the share of *Negativibacillus* has been previously identified in patients with UC [64], and an increase in the proportion of *Mogibacterium* has been associated with a higher risk of colorectal cancer [65].

It is noteworthy that the initial microbiota of patients who had no improvements after FMT substantially differed from the microbiota of other patients. In patient #6, the initial diversity was significantly reduced and many bacterial genera that were found in other patients were absent in the microbiota of this patient. As a result of FMT, the diversity of the microbiota substantially increased, both due to bacteria that have a beneficial effect on the human intestine (such as *Roseburia* and *Asteroleplasma* [66,67]) and potentially pathogenic bacteria (genera *Oscillospiraceae*, *Vibrio*, *Veillonella*, *Collinsella*). It has been previously shown that bacteria of the genus *Oscillospiraceae* are able to break down mucin, a basic polysaccharide of the mucus lining the intestinal wall [68], whereas members of the genus *Veillonella* stimulate the production of inflammatory cytokines [69]. However, before FMT, this patient had a significantly increased proportion of the genera *Parvimonas* and *Aggregatibacter*, for which a positive correlation with the dysbiotic state of the intestine has been previously found [70,71]. After FMT, the number of sequences belonging to these bacteria was reduced.

As for patient #14, the microbiota was substantially more diverse before FMT than in other patients. Bacteria of the genera *Agathobacter*, *Stenotrophomonas*, *Aliidiomarina*, *Sediminibacterium*, and *Noviherbaspirillum* were more prevalent in the microbiota of this patient and the last two genera were found only in this patient. The role of these bacteria in the human intestine is still not clear. Also, this patient had an increased share of unclassified bacteria and bacteria of the genera *Acinetobacter* and *Campylobacter* before FMT. According to the published data, members of these genera can cause inflammatory bowel disease in humans [72,73]. It should be noted that after FMT, the microbiota diversity of this patient decreased, as did the prevalence of all the listed bacteria. At the same time, after the procedure, an increase in the number of bacteria of the genus *Bilophila* was detected, for which the ability to cause chronic inflammation in mice has been previously shown [74].

The initial microbiome of the non-responder (patient #13), whose condition worsened after FMT, was enriched in the sequences of the *Dialister*, *Vibrio*, *Collinsella*, and *Haemophilus* genera compared to other patients. The share of these bacteria was substantially reduced after FMT and an increase in the *Prevotella* and *Veillonella* genera was observed. The controversial role of bacteria from the *Prevotella* genus has been previously shown [75]. Members of this genus are among the most abundant human gut commensals; however, an ability to degrade the mucus layer has been found, which confirms their possible pro-inflammatory role [75]. 

## 4. Discussion

In this study, the efficacy of a series of FMT enemas was assessed in patients with UC with mild and moderate disease activity. Importantly, before FMT most patients received preparations with 5-aminosalicylate and/or other preparations. However, they did not achieve the desired improvement. After FMT, 95% of patients achieved a clinical response at week 8 that indicated that the used FMT procedure was tolerated and safe. Only one non-responder of twenty patients was recorded after FMT, and at least in two patients the improvement did not last long (≥8 weeks). The periods of improvement were different: two-thirds of the interviewed patients reported remission for ≥6 months, more than a third of patients had remission that lasted >18 months, and four patients (31%) had remission of more than two years. Surprisingly, one patient reported an improvement over 4 years, which continues at the present time. Notably, the duration severity of the initial condition of patients did not affect the duration of improvement.

The results of several studies on the effectiveness of FMT in UC have been previously published [18,19,20,21,22], including our study in which patients with UC were given only one FMT enema with material from one donor [76]. In this study, the remission rate is comparable to that reported by Březina et al. [22] and higher than those in the other studies [18,19,20,21,76]. On the one hand, our result can be explained by a sampling of patients with only mild and moderate UC. On the other hand, material from several donors was used for each FMT enema, which ensured the introduction of a more diverse microbiota.

The results of 16S rRNA profiling of bacterial communities indicated a significant heterogeneity in the biodiversity and taxonomic composition of the gut microbiota of patients with UC, which is consistent with the multifactorial pathogenesis of this disease [77]. Despite the fact that the average biodiversity of microbial communities in patients with UC was reduced to that in healthy volunteers, this association was not universal for all patients. Thus, a decrease in the Shannon index after FMT was observed in several patients that had high initial biodiversity and the decrease in diversity after FMT could be caused both by the active reproduction of potentially invasive bacteria (as in the case of *Fusobacterium* spp. in patients #2, #3, #6, #7, and #14) and by the elimination of pathogenic species. This could lead to a lack of correlation between changes in the microbiota biodiversity and the condition of patients after the procedure.

Notably, the high heterogeneity of the taxonomic composition at low taxonomic levels characterized all intestinal microbial communities, and it was observed not only in patients with UC, but also in healthy people. However, a distinctive characteristic of the microbiomes of patients with UC was an increased proportion of various potentially pathogenic bacteria compared to those in healthy volunteers. It is not known whether these bacteria play a role in the pathogenesis of UC, or the increase in their abundance is a consequence of the disease. Nevertheless, a decrease in their proportion after FMT may be one of the factors that positively affect the condition of patients. In addition, we observed an increase in the proportion of bacteria producing beneficial metabolites after FMT, which may also be an important factor in reducing the severity of the disease symptoms.

Our data confirmed the ambiguous effect of the FMT on the condition of patients with UC. Different effects of FMT on the patients’ condition could be a consequence of different initial compositions of the microbiota. Thus, both the non-responder and patients who reported no improvement 12 weeks after FMT had significant deviations in the compositions of their microbiota from other patients. However, the nature of these deviations was different, which does not allow us to reveal a correlation between a certain microbiota composition and the potential effectiveness of FMT in patients with UC. In addition, FMT is always associated with the risk of introducing potentially invasive bacteria into the patient’s intestines. Despite the fact that all donors were tested for the most common pathogenic bacteria, viruses, and helminths, there are microorganisms that are not pathogens but can cause a pro-inflammatory effect on the human intestine.

This study had some limitations. First, patients with different severities of UC were involved in the study. Second, the samplings of patients and volunteers differed in age and gender composition. Therefore, the comparison of microbiomes between these samples is not entirely correct. The main limitation is that we could not examine patients for longer than eight weeks, since the COVID-19 pandemic began and patients could not come for checkups.

In conclusion, this study confirmed the efficacy of FMT enemas in the treatment of UC, which might be associated with a significant increase in gut microbiome diversity and normalization of microbiota composition. Nevertheless, the effect of the procedure was ambiguous, probably due to different initial baseline characteristics of the patients. A possible way to increase the FMT efficiency is an improvement in the donor selection, using verified material from several donors for one FMT procedure, and dedicated analysis of the patients’ microbiome before FMT. Taking into consideration the substantial difference between the microbiota of healthy people, a personalized approach should be applied by selecting donors for the patient in accordance with their specific microbiota. Furthermore, other methods of influencing the intestinal microbiota that do not use the introduction of live bacteria are currently being tested, for example, the application of sterile stool filtrate from healthy donors.

## Figures and Tables

**Table 1:** Patient baseline characteristics.

Male		11 (55%)
Female		9 (45%)
Age		36 (25–65)
Disease duration (years)		4 (1–12)
Total Mayo score	3–5	19 (95%)
	6	1 (5%)
Fecal calprotectin (µg/g)		750.5 (122–2000)
C-reactive protein (mg/L)		5.1 (0.43–39.4)
Hemoglobin (g/L)		133 (87–167)
Used therapy	5-aminosalicylate	18 (90%)
	Steroids	4 (20%)
	Immunomodulator	3 (15%)
	Antispasmodic	1 (5%)

The data provide the number of patients (%) or median (range).

**Table 2:** Duration of improvement.

Time Period	Clinical Outcome	Number of Patients (%)
Week 2	Clinical response	19 (95%)
Week 8	Clinical remission	12 (60%)
	Clinical response	19 (95%)
Week 12	Clinical remission	9 from 13 interviewed patients (69%)
	Clinical response	11 from 13 interviewed patients (85%)
Month 6	Clinical remission	9 * from 13 interviewed patients (69%)
Month 12	Clinical remission	5 * from 13 interviewed patients (38%)
Month 18	Clinical remission	5 * from 13 interviewed patients (38%)
Month 24	Clinical remission	4 * ** from 13 interviewed patients (31%)

* One of the patients achieved only clinical response at week 2. ** These patients reported clinical remission 48 months after FMT.

**Table 3:** Results of sequencing libraries of 16S rRNA gene fragments; N—the number of reads.

Group	N_average_, (N_min_–N_max_) before Filtration	N_average_, (N_min_–N_max_) after Filtration	Number of OTU
HV	114,670, (95,892–156,040)	59,601, (33,893–93,798)	1794
UC-bef	139,819, (83,382–175,968)	51,660, (22,621–81,037)	1818
UC-aft	142,060, (107,889–218,576)	54,773, (32,919–105,051)	1959

**Table 4:** Average proportions of the main bacterial classes in samples from healthy volunteers (HV) and patients with UC before (UC-bef) and after (UC-aft) FMT.

Phylum	Class	Average Proportion, %		
		HV	UC-bef	UC-aft
Firmicutes	Clostridia	64.7	46.7	42.4
	Negativicutes	7.7	5.7	4.0
	Bacilli	0.8	1.3	2.8
Bacteroidota	Bacteroidia	26.0	34.2	34.0
Proteobacteria	Alphaproteobacteria	0.2	5.2	2.8
	Gammaproteobacteria	1.5	3.9	4.6
Fusobacteriota	Fusobacteriia	0.002	0.06	7.2
Actinobacteriota	Actinobacteria	0.7	0.46	1.1
	Coriobacteriia	0.07	0.73	0.77

## Data Availability

Data are contained within the article and Supplementary Materials.

## Supplementary Materials

The following supporting information can be downloaded at: https://www.mdpi.com/article/10.3390/jcm12247702/s1, Figure S1: Taxonomic classification of OTUs at the family level based on the Silva v.132 (full) database; Figure S2: Taxonomic classification of OTUs at the genus level based on the Silva v.132 (full) database; Table S1: The condition of patients with UC before and after FMT according to the survey; Table S2: Shannon indices for patients before and after FMT.
