# Supplementary material for: Intestinal Microbiome Changes and Clinical Outcomes of Patients with Ulcerative Colitis after Fecal Microbiota Transplantation
Source: J Clin Med. 2023 Dec 15;12(24):7702. doi: 10.3390/jcm12247702 (PMC10743744; doi:10.3390/jcm12247702)
Supplement: Supplementary file 1 [file jcm-12-07702-s001.zip › Supplementary Tables.pdf]

Table S1. The condition of patients with UC before and after FMT according to the survey.

Patient	Sex	Age	Clinical remission or re response at week 8	Improvements after FMT (interview)	Total duration of improvements, months	Current number of defecations per day*		Presence of blood in the stool at the time of survey*		Suggested cause of recurrence	Supporting medications used
						Before FMT	After FMT	Before FMT	After FMT		
**1**	F	29	rem	+	18**	2-3	1-2	+	-		Mesalazine
**2**	F	42	rem	+	6	1-2	1-2	+	-	Severe stress	Azathioprine, Mesalazine
**3**	M	57	rem	+	24	5	2-3	+	-	Surgery (cholecystostomy)or treatment of pneumonia with a triple course of antibiotics	
**4**	M	34	res	+	48**	7-8	Up to 3	-	-		Sulfasalazine, Mesalazine
**6**	F	34	res	-	2	4	2	+	+		Mesalazine
**8**	M	42	rem	+	6**	2-3	1	+	-		Mesalazine
**9**	F	25	rem	+	6**	1-2	1-2	-	-		Mesalazine, Kolofort, Prednisolone, 6-Mercaptopurine, Zakofalk
**12**	M	36	rem	+	24**	1	1-2	-	-		Mesalazine
**14**	F	34	res	-	2	2-8	Up to 16	+	+		Mesalazine, Zakofalk
**15**	M	46	res	+	4	1	1	+	-		
**17**	M	36	rem	+	4	1	1	-	+	Diet violation	Mesalazine
**19**	F	26	rem	+	24**	Up to 4	1	+	-		Mesalazine, Trimedat
**20**	M	34	rem	+	6	Up to 5	2-12	+	+	Preparation for sigmoidoscopy	GCS, Mesalazine

* At the time of the survey. ** No recurrences of the disease at the time of the survey.

Table S2. Shannon indices for patients before and after FMT

Patient	Shannon index before FMT	Shannon index after FMT
**1**	5.03	3.92
**2**	6.16	3.40
**3**	5.90	5.76
**4**	2.77	5.94
**5**	3.27	3.37

**6**	3.28	5.10
**7**	5.03	3.33
**8**	5.82	6.14
**9**	3.84	6.49
**10**	3.08	5.62
**11**	4.86	5.57
**12**	4.44	5.63
**13**	4.72	5.77
**14**	5.76	5.55
**15**	4.83	6.16
**16**	5.23	5.78
**17**	4.63	5.94
**18**	4.88	4.92
**19**	4.87	5.59
**20**	5.77	6.49
