# Supplementary material for: Intestinal Microbiome Changes and Clinical Outcomes of Patients with Ulcerative Colitis after Fecal Microbiota Transplantation
Source: J Clin Med. 2023 Dec 15;12(24):7702. doi: 10.3390/jcm12247702 (PMC10743744; doi:10.3390/jcm12247702)
Supplement: Supplementary file 1 [file jcm-12-07702-s001.zip › Figure S1.pdf]

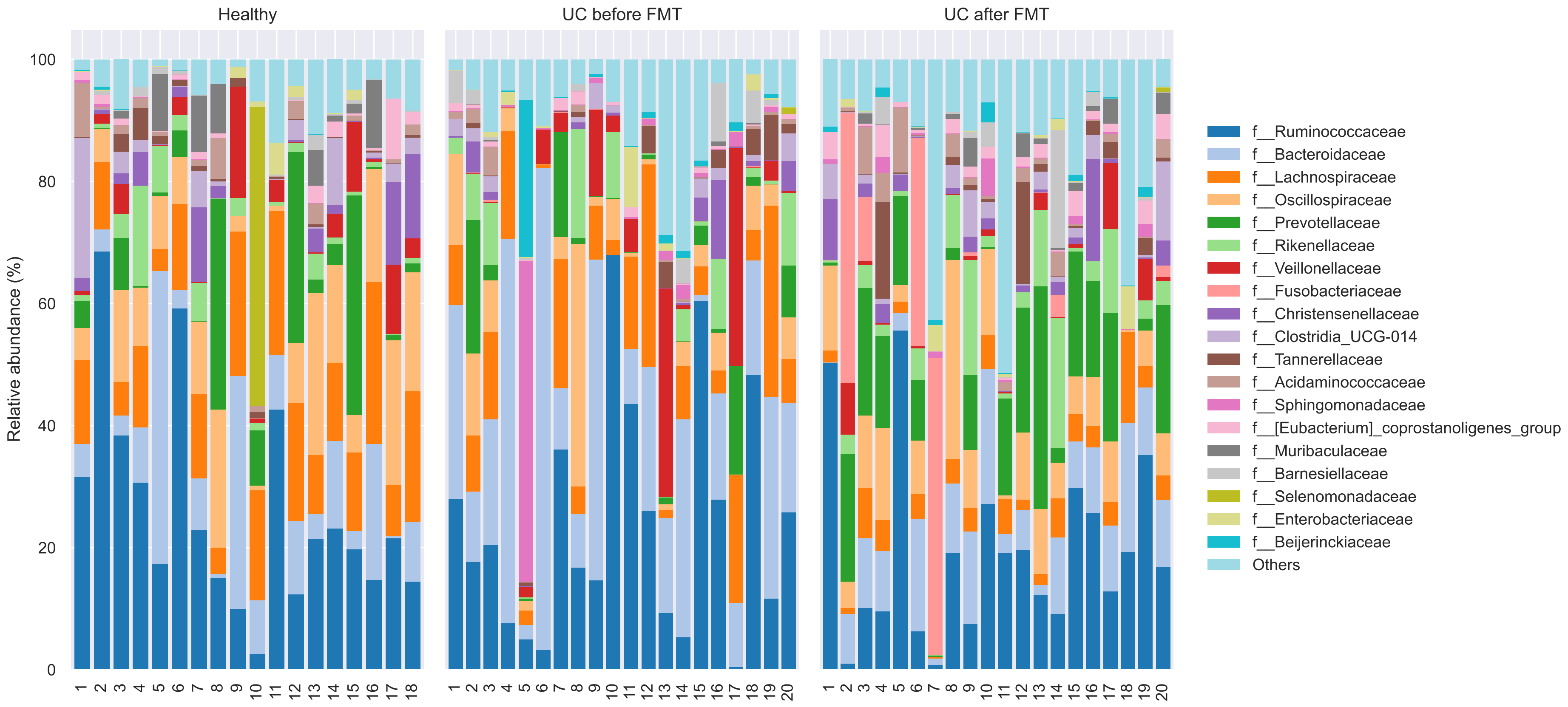

Figure S1. Taxonomic classification of OTUs at the family level based on the Silva v.132 (full) database.
